# Cure of Cancers

**Published:** 1849-01

**Authors:** 


*Cure of Cancers.*—In the summer of 1847, when travelling in Western Virginia, we gave an account of the (apparently) permanent cure, by the application of some kind of caustic, to the breast of a lady, which had been regarded as affected with fungus hæmatodes. Eight months had then elapsed and the new integuments appeared perfectly sound. The caustic was applied by a physician of Galliopolis.

The account of this case having been seen by the friends of a lady of this city, whose right breast was in a state of schirrus advancing to ulceration, she determined to place herself in the care of a Cincinnati partner of the Galliopolis practitioner. In five or six weeks, his applications had caused an entire sloughing of the gland, and complete cicatrization followed. On the lapse of a few months, however, the new integuments assumed a well marked carcinomatous aspect, and at this time are in a state of cancerous ulceration. The disease is, also, advancing in the other breast, and the arm of the originally affected side, is in a semi-œdematous condition, and the seat of a great deal of pain. Having, by the publication of the other case, given a sort of currency to the practice by which it was cured, we consider it a duty to make known the failure of the same method in this case; in doing which we feel bound to add that there was probably a mistake in the diagnosis of the first case, which might not have been of a malignant character.—*The Western Journal of Medicine and Surgery.*

